# Editorial: Autophagy and Related Transcription Factors in Liver and Gut Diseases

**DOI:** 10.3389/fphar.2019.01610

**Published:** 2020-01-30

**Authors:** Nabil Eid, Manoj B. Menon, Paul Thomes, Tao Zeng, Nuno Raimundo, Jose C. Fernandez-Checa, Lin Wang, Yuko Ito, Yoshinori Otsuki, Ernest Adeghate

**Affiliations:** ^1^Department of Anatomy, College of Medicine and Health Sciences, United Arab Emirates University, Al Ain, United Arab Emirates; ^2^Kusuma School of Biological Sciences, Indian Institute of Technology Delhi, New Delhi, India; ^3^Departments of Internal Medicine and Biochemistry & Molecular Biology, University of Nebraska Medical Center, Omaha, NE, United States; ^4^Institute of Toxicology, School of Public Health, Shandong University, Jinan, China; ^5^Institute for Cellular Biochemistry, University Medical Center Göttingen, Göttingen, Germany; ^6^Department of Cell Death and Proliferation, Institute of Biomedical Research of Barcelona (IIBB)-CSIC, IDIBAPS and CIBEREHD, Barcelona, Spain; ^7^Research Center of ALPD, Keck School of Medicine, University of Southern California, Los Angeles, CA, United States; ^8^College of Animal Science and Veterinary Medicine, Shandong Agricultural University, Tai'an, China; ^9^Department of General and Gastrointestinal Surgery, Osaka Medical College, Takatsuki, Japan; ^10^Osaka Medical College, Takatsuki, Japan

**Keywords:** autophagy, diabetes, ethanol, lipophagy, liver, mitophagy, pancreas, steatosis

The cell biologist Yoshinori Ohsumi received the 2016 Nobel Prize in Medicine for his early identification and characterization of the autophagy machinery, in particular, AuTophaGy-related (Atg) genes, in yeast. Macroautophagy (hereafter, autophagy) is a cytoprotective pathway for sequestration of cellular components (such as misfolded proteins, damaged organelles, and excessive lipids) into autophagosomal vesicles, followed by clearance *via* the lysosomal system (Galluzzi et al., 2017). Autophagy is specifically upregulated upon exposure to various stressors such as oxidative and endoplasmic reticulum stress, thus aiding in the prevention of various pathologies. Therefore, autophagy dysregulation may be involved in inflammatory, metabolic, toxic, and infectious diseases and cancer (Kroemer et al., 2010; Eid et al., 2013; Horibe et al., 2017). Most organelles also seem to have selective programs of autophagy, including mitochondria, lipid droplets, endoplasmic reticulum, and even lysosomes. Selective autophagic removal of damaged mitochondria, or mitophagy, is an anti-apoptotic mechanism induced and specifically upregulated in response to various damaging agents such as binge ethanol exposure or drug-induced liver injury in animal models (Otsuki et al., 1994; Youle and Narendra, 2011; Lemasters, 2014; Eid et al., 2016; Eid et al., 2019). Autophagy can be regulated not only at the gene level, but its final performance can be modulated by lysosomal lipid composition. For instance, accumulation of lipids (e.g., cholesterol) in lysosomes has been shown to impair the fusion of autophagosomes (containing disrupted mitochondria) with lysosomes, contributing to the perpetuation of damaged mitochondria, which sensitizes to acetaminophen hepatotoxicity (Baulies et al., 2015). On the other hand, autophagic clearance of lipid droplets is referred to as lipophagy (Singh and Cuervo, 2012). Various transcription factors such as transcription factor EB (TFEB), Nrf2, HIF, and Foxo3a play important roles in the regulation of autophagy and mitophagy-related proteins such as LC3, cathepsins, and Parkin (Sardiello, 2016; Horibe et al., 2017; Eid et al., 2019). The focus of this Research Topic is to highlight the involvement of these transcription factors in the regulation of liver and gut diseases through autophagy pathway as these are potential therapeutic targets for the restoration of autophagy and in the management of these diseases.

This Research Topic compiles nine articles, including four reviews and five original research contributions. The interesting review by Su et al., on *Mitophagy in Hepatic Insulin Resistance: Therapeutic Potential and Concerns*, focuses on advances in the understanding of relationship between mitophagy and hepatic insulin resistance and the potential value of mitophagy in the treatment of hepatic insulin resistance and metabolic syndrome (*via* clearance of damaged mitochondria and subsequent reduction of lipid accumulation). This observation is supported by an elegant study demonstrating that loss of Parkin-mediated mitophagy promoted further β-cell failure under pathological stress conditions including STZ exposure and leptin receptor defects (Hoshino et al., 2014).

Recent advances with incretin-associated drugs have opened new avenues in the management of diabetes. In another interesting review article, Kanasaki et al. analyzes distinct molecular mechanisms of autophagy regulation by glucagon, GLP-1, and DPP-4 inhibitor. In addition, they also discuss the potential contribution of these regulatory pathways in the induction of beneficial autophagy-upon bariatric surgery, which have implications in the treatment of diabetic diseases (Adeghate et al., 2019).

Lipophagy, a process controlled by the autophagy master regulator, TFEB, is key to maintaining a healthy liver. The third review by Yang et al. discusses the different lipophagic responses in rodent hepatocytes after exposure to acute and chronic ethanol. They showed that these responses are controlled by subcellular TFEB localization. They suggest that natural products and drugs such as caffeine/coffee, resveratrol, corosolic acid, zinc, carbamazepine, and rapamycin may activate autophagy/lipophagy for preventing or even aiding in the treatment of alcohol-induced fatty liver. In addition, they stress that the specific upregulation of TFEB by certain small molecules (related to digoxin, ikarugamycin, and alexidine dihydrochloride) may be of therapeutic value in the treatment of human fatty liver disease (Wang et al., 2017).

In another review article, Zhang L. et al. elegantly summarize the current understanding on the use of herbal medicine extracts and natural products for activation of hepatic autophagy, thus helping in the prevention and treatment of non-alcohol fatty liver diseases (NAFLD). A specific focus is set on mechanisms by which autophagy can target the main events in the pathogenesis of NAFLD, including hepatic steatosis, inflammation, oxidative stress, and apoptosis.

The research article by Fan et al. provides novel data supporting a protective role for methylprednisolone (MP) in an experimental autoimmune hepatitis (AIH) model, possibly mediated by the Akt/mTOR signaling pathway. MP seems to ameliorate apoptosis and promote autophagy in hepatocytes in *in vitro* and *in vivo* mouse model. They suggest a potential use of MP to treat AIH. Their study provides interesting insights into the mechanisms underlying the effect of MP on hepatocytes.

The interesting study by Guo et al. explores the effects of 6-bromo-indirubin-3′-oxime (6BIO), a potent inhibitor of glycogen synthase kinase-3 (GSK-3), on the aging rodent liver. They found that 6BIO mitigates oxidative stress, improves lipid metabolism, enhances autophagy, and significantly retards liver aging *via* modulating the GSK-3β and mTOR pathways. They suggest that 6BIO could be a potential agent to protect the liver in the field of anti-aging pharmacology.

Hepatitis C virus (HCV) dysregulates lipid metabolism to accomplish several steps of its life cycle (Paul et al., 2014; Strating and van Kuppeveld, 2017). Vescovo et al. investigates the impact of mevastatin (a cholesterol-lowering agent isolated from *Penicillium citinium*) on HCV replication and autophagy in MMHD3 non-transformed hepatocytes harboring sub-genomic HCV replicons, specifically in relation to the extracellular lipid uptake. In contrast to the previous studies in transformed human cell lines, they observed drastic upregulation of intracellular cholesterol in MMHD3 cells upon mevastatin treatment, which is associated with enhanced lipophagy and HCV replication. However, these effects are reversed when cells are cultured in delipidated serum, which establishes the fact that suppression of extracellular lipid uptake is as important as inhibiting cholesterol biosynthesis in suppressing HCV replication. This study may have implications in the development of treatment modalities targeting cholesterol levels to limit HCV replication.

Fan et al., in their original research article, report on isoorientin-mediated suppression of APAP-induced hepatotoxicity in mice *via* activation Nrf2 anti-oxidative pathway and the involvement of AMPK/Akt/GSK3β signaling. This hepatoprotective effect of isoorientin could be mediated by autophagy activation, as reported by others (Muhammad et al., 2018; Lv et al., 2019). The last article by Zhang et al. conclude that salvianolic acid B inhibits activation of human primary hepatic stellate cells through downregulation of MEF2 (myocyte enhancer factor 2) signaling pathway, resulting in subsequent amelioration of stellate cell-mediated hepatic fibrosis. However, we cannot rule out the possible involvement of autophagy in the hepatoprotective effect of salvianolic acid B as it has been reported that autophagy may be required for stellate cell activation and hepatic fibrosis in alcohol liver disease (Eid et al., 2013).

The field of autophagy research is growing at a rapid pace and the discoveries revealing novel roles for the autophagy pathway in diverse pathologies are making it a very attractive target for pharmacological intervention. Strategies are being envisaged for therapeutic upregulation and/or suppression of autophagy and/or specialized processes like lipophagy or mitophagy. The collection of articles in this Research Topic, including original research and reviews, are aimed at summarizing some of these ideas within the specialized field of gastrointestinal/hepatic pharmacology and beyond.

## Author Contributions

All the authors contributed to this editorial work.

## Funding

We acknowledge support from grants SAF-2015-CIBEREHD,and by AGAUR SGR-2017-1112.

## Conflict of Interest

The authors declare that the research was conducted in the absence of any commercial or financial relationships that could be construed as a potential conflict of interest.
